# Multilayered Nanocarriers as a New Strategy for Delivering Drugs with Protective and Anti-inflammatory Potential: Studies in Hippocampal Organotypic Cultures Subjected to Experimental Ischemia

**DOI:** 10.1007/s12035-024-04670-y

**Published:** 2025-01-09

**Authors:** Kinga Kamińska, Beata Grygier, Magdalena Regulska, Magdalena Procner, Monika Leśkiewicz, Marta Szczęch, Juan Yang, Aud Bouzga, Piotr Warszyński, Władysław Lasoń, Krzysztof Szczepanowicz, Agnieszka Basta-Kaim

**Affiliations:** 1 Laboratory of Immunoendocrinology Department of Experimental Neuroendocrinology, Maj Institute of Pharmacology, Polish Academy of Sciences, 12 Smętna St, 31-343 Kraków, Poland; 2 Jerzy Haber Institute of Catalysis and Surface Chemistry, Polish Academy of Sciences, Niezapominajek 8, 30-239 Kraków, Poland; 3 SINTEF Material and Chemistry, Forskningsveien 1, NO-0314 Oslo, Norway

**Keywords:** Hippocampal organotypic cultures, Ischemia, AOT nanoparticle, PCL nanoparticle, Carnosic acid-free and encapsulated, Neuroprotection, Neuroinflammation

## Abstract

Oxidative stress and neuroinflammation play a pivotal role in pathomechanisms of brain ischemia. Our research aimed to formulate a nanotheranostic system for delivering carnosic acid as a neuroprotective agent with anti-oxidative and anti-inflammatory properties to ischemic brain tissue, mimicked by organotypic hippocampal cultures (OHCs) exposed to oxygen–glucose deprivation (OGD). In the first part of this study, the nanocarriers were formulated by encapsulating two types of nanocores (nanoemulsion (AOT) and polymeric (PCL)) containing CA into multilayer shells using the sequential adsorption of charged nanoobjects method. The newly designed nanoparticles possessed favorable physicochemical characteristics as reflected by zeta potential and other parameters. Next, we demonstrated that the newly designed gadolinium-containing nanoparticles were not toxic to OHCs and did not affect the detrimental effects of OGD on the viability of the hippocampal cells. Importantly, they readily crossed the artificial blood–brain barrier based on the human cerebral microvascular endothelial (hCMEC/D3) cell line. Furthermore, the PCL-Gd carnosic acid–loaded nanoparticles displayed anti-inflammatory potential, expressed as decreased OGD-induced HIF-1α and IL-1β levels. Results of the molecular study revealed a complex mechanism of the nanoformulation on ischemia-related neuroinflammation in OHCs, including anti-inflammatory protein A20 stimulation and moderate attenuation of the NFκB signaling pathway. Summing up, this study points to acceptable biocompatibility of the newly designed CA-containing theranostic nanoformulation and emphasizes their interaction with inflammatory processes commonly associated with the ischemic brain.

**Graphical Abstract:**

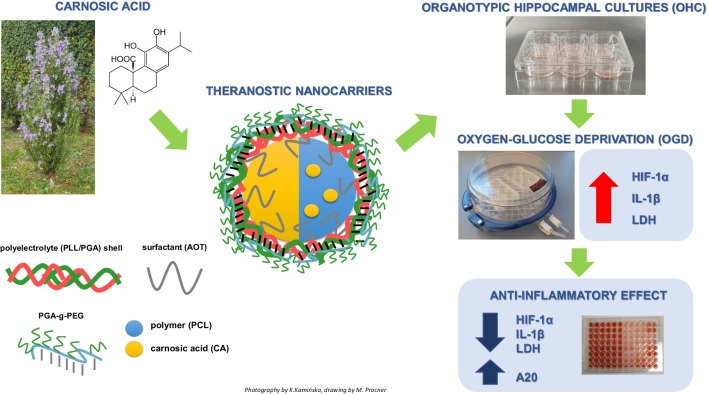

## Introduction

Ischemic stroke is one of the leading causes of death and disabilities worldwide. The sudden loss of blood circulation to an area of the brain results in neurologic deficits and the initiation of numerous pathological processes such as oxidative stress, excitotoxicity, hypoxia, cerebral edema, and inflammation. They may lead to primary neuronal death in the ischemic areas of the brain, within a short time after brain damage likely due to necrosis, while the delayed neuronal death occurring over days and months after stroke bears features of an apoptotic process [1]. The occurrence of delayed cell death creates favorable conditions for a therapeutic window, giving a chance for timely pharmacological intervention. The current treatment options for stroke include thrombolysis, anticoagulation, antihypertensive, plasmin reduction, and catheter intervention. However, despite extensive research, no neuroprotective drug has been marketed so far. A substantial number of potential neuroprotective drugs failed preclinical and clinical trials due to their unfavorable pharmacodynamics and pharmacokinetic properties, especially low bioavailability, poor water solubility and stability, and insufficient ability to cross the blood–brain barrier. To this end, nanotechnology offers an advanced strategy for improving drug delivery to stroke-affected brain regions using nanocarriers. Regarding polymeric nanocarriers, among the various encapsulation methods, the sequential adsorption of charged nanoobjects, also known as the layer-by-layer (LbL) method, merits attention [2–4]. The advantage of the LbL method is multifunctionality that comes from the possibility of the formation of the multilayer shell by numerous functional charged species, e.g., antibodies, aptamers, or inorganic nanoparticles [5–8].

The theranostic drug carriers are not only able to deliver their encapsulated cargo to the brain but the delivery can be monitored by some imaging technique for optimizing targeting and dosage. Nevertheless, visualization of the uptake of nanoparticles used as drug carriers into the brain using magnetic resonance imaging (MRI) requires sensitization with specific approaches like SPION methods, paramagnetic complexes (e.g., gadolinium (Gd), dysprosium (Dy), europium (Eu)), or molecules containing 19F atoms. Since some contrast agents may have a toxic effect [9], therefore, the in vivo testing of newly designed theranostic drug delivery systems should be preceded by rigorous evaluation of its effect on cell viability in vitro and ex vivo.

The present study proposes a new method of synthesizing multilayered nanocarriers (NCs) with emulsion and polymeric core, also sensitized with gadolinium as a potential theranostic delivery system for targeting ischemic/reperfusion pathology. First, the physicochemical characteristics of nanoparticles, including size, shape, zeta potential, and stability, have been determined. Since multicore NCs are dedicated to intravenous administration, we evaluated the ability of the NCs to cross the blood–brain barrier using the human cerebral microvascular endothelial (hCMEC/D3) cell line in the basal and oxygen-glucose deprivation (OGD) model. All next experiments were performed in organotypic hippocampal cultures (OHCs), a widely accepted ex vivo model in which the morphological structure and functional interactions between cells in the brain are preserved, mimicking in vivo conditions. We estimated the impact of new NCs on the cell viability, nitric oxide release, and HIF-1α protein level, as well as the effect of NCs on pro- and anti-inflammatory parameters under basal and ischemia/reperfusion conditions.

As a biologically active substance for encapsulation in the newly designed NCs, we chose carnosic acid (CA). CA is a polyphenol extracted mainly from *Rosemarinus officinalis*. It belongs to the labdane type of diterpenes of the 20-carbon skeleton [10], and its phenolic structural moiety is common in flavonoids and other aromatic compounds of the shikimic acid and acetate metabolic pathways in plants. A broad array of biological effects of CA including anti-oxidative and anti-inflammatory activities have been well documented [11]. Specifically, CA was reported to reduce neuronal cell death through activation of the Nrf2/ARE transcriptional pathway, which leads to the upregulation of endogenous antioxidant enzymes [12]. Since oxidative stress and neuroinflammation are crucially involved in the pathology of ischemic stroke, CA may be considered as a potential drug for, at least, supportive treatment of this neurologic disorder. Indeed, the recent in vitro study demonstrated that CA has favorable neuroprotective effects in models of oxidative stress–induced neuronal damage [13].

However, although CA readily crosses the blood–brain barrier (BBB) [14], its utility as a potential neuroprotective drug is hampered by its poor water solubility and instability. Therefore, in the present study, the utility and the mechanism of action of CA, both in free and loaded in nanoparticle forms in the experimental model of ischemia/reperfusion, was also determined.

## Materials and Methods

### Chemicals

Carnosic acid was received from ChemNorm Biotech Co., Ltd. Polyelectrolytes used to prepare multilayered shells of nanocarriers were as follows: poly-l-lysine hydrobromide (PLL, M_w_ 15,000–30,000), poly-l-glutamic acid sodium salt (PGA, M_w_ 15,000–50,000) both from Sigma–Aldrich, gadolinium-labelled poly-l-lysine (PLL-Gd, M_w_ 22,000) from BioPAL, pegylated polyanion PGA-g-PEG (g ~ 30% and PEG, M_w_ 5000), and rhodamine-labeled polycation PLL-Rod were synthesized according to the protocol described previously [15, 16]. Docusate sodium salt (AOT), polycaprolactone (PCL, M_w_ 14,000), chloroform, and sodium chloride were obtained from Sigma-Aldrich. Ethyl alcohol (99.8%) was purchased from Avantor Performance Materials Poland S.A., while ultra-purified water was obtained using the Direct-Q5 UV purification system from Millipore (Warsaw, Poland). All chemicals and solvents were of analytical grade and used as received.

### Preparation of Polyelectrolyte Multilayer Nanocarriers

Multilayered nanocarriers with two types of core (polymeric and nanoemulsion ones) were formulated by previously developed methods [17–20]. Polymeric and nanoemulsion cores loaded with carnosic acid were formed by the spontaneous-emulsification solvent evaporation (SESE) method. The oil phase based on easily evaporative organic solvent chloroform contains carnosic acid (100 mg/ml), AOT (330 mg/ml), and in the case of polymeric core PCL (10 mg/ml), while the aqueous phase contains PLL (0.2 mg/ml) and NaCl 0.09%. The nanoemulsions were formed by the addition of 0.1 ml of oil phase mixed with 10 ml of absolute ethanol to 200 ml of agues phase during continuous stirring. The polymeric or nanoemulsion cores were formed after the evaporation of the organic solvents by continuous stirring, where the final concentration of chloroform did not exceed 0.04 mg/l [15]. Such formulated cores were encapsulated into a multilayered shell via a layer-by-layer technique [2–4] with the following polyelectrolytes: PLL, PGA/ PLL-Gd, PLL-Rod, PGA-g-PEG.

### Determination of Particle Size and Zeta Potential and Encapsulation Efficacy

The hydrodynamic size and zeta potential were determined from dynamic light scattering measurements and electrophoretic mobility, respectively, using Zetasizer Nano ZS, Malvern Panalytical Instruments, UK. All measurements were performed at 25 °C in 0.09% NaCl, and each value was received as an average of at least three subsequent measurements with 20 runs. The nanocarriers were analyzed also by a cryo-scanning electron microscope (cryo-SEM) Jeol JSM-7600F Field Emission Scanning Electron Microscope (FESEM) (Jeol Ltd., Tokyo, Japan). Confirmation of encapsulation and determination of encapsulation efficacy were based on UV–vis spectrometry measurements by UV-1800 spectrophotometer, Shimadzu Corporation, Kyoto, Japan.

### Animals

Sprague–Dawley rats (226–275 g) were purchased from Charles River (Sulzfeld, Germany) and maintained under conventional laboratory conditions at a temperature of 23 °C, with a 12-h light/12-h darkness cycle (commencing at 08:00), and provided *ad libitum* access to water and food. Following an acclimatization period, the stage of the estrous cycle in the female rats was determined by measuring vaginal wall impedance (model MK-12UB, Ugo Basile, Gemonio, Italy). On the day of proestrus, female rats were paired with males for 12 h, and the presence of sperm in vaginal smears was assessed the subsequent morning. All experimental procedures adhered to the guidelines stipulated by the Committee for Laboratory Animal Welfare and Ethics of the Maj Institute of Pharmacology, Polish Academy of Sciences, Krakow, Poland. The protocol for generating the organotypic hippocampal cultures (OHCs) is in line with the European Union (Directive 2010/63/EU, amended by Regulation (EU) 2019.1010) guidelines on the ethical use of animals and according to national regulations. All experiments were conducted according to the principles of the Three Rs, and all efforts were undertaken to minimize the usage of animals and alleviate any potential suffering.

### Organotypic Hippocampal Cultures

OHCs were established by the Stoppini et al. [21] protocol, incorporating slight modifications as described by Bryniarska-Kubiak et al. (2023) and Tylek et al. (2023) [22, 23]. The cultures were prepared from 6- to 7-day-old Sprague Dawley pups. After decapitation, the isolated brains were immediately placed into a sterile ice-cold working buffer consisting of 96% HBSS, 3.5% glucose, and 0.5% penicillin/streptomycin (all reagents obtained from Gibco, Waltham, MA, USA). The hippocampi were isolated and placed on Teflon disks, and sliced into 350-µm-thick sections using a McIlwain™ Tissue Chopper (Surrey, UK). Subsequently, selected sections were transferred into ThinCerts™ inserts with 0.4-μm pore size membranes (Greiner Bio-one, Kremsmunster, Austria) in 6-well plates containing 1 mL of initial medium enriched with 25% horse serum (50% DMEM + GlutaMax™-I, pH 7.4; 20.5% HBSS; 25% horse serum; 0.1 mg/mL glucose; 1% amphotericin B; 0.4% penicillin and streptomycin; 1% B-27 supplement; and HEPES—all reagents obtained from Gibco, London, UK). OHCs were maintained in an incubator at 37 °C under an atmosphere of 95% air and 5% CO_2_. The culture medium was changed every 48 h, with a gradual reduction in horse serum concentration from day 4 to day 7. By the 7th day, the medium became serum-free and was composed of 50% DMEM F-12, 44% HBSS, 0.1 mg/mL glucose, 1% B-27 supplement, 1% N2 supplement, 1% amphotericin B, 0.4% penicillin and streptomycin, and HEPES.

### Oxygen–Glucose Deprivation

The OGD procedure was conducted on the 7th (OHCs) or 5th (BBB model) day after the culture establishment, following the Bryniarska-Kubiak et al. (2023) [22] protocol. OHCs or hCMEC/D3 cells were washed twice in Ringer’s solution containing 10 mM mannitol (Sigma–Aldrich, St. Louis, MO, USA). Subsequently, the membranes with OHCs or hCMEC/D3 cells were relocated to new 6- or 24-well plates respectively containing 1 mL or 300 µl Ringer’s solution and transferred into a hypoxic chamber (maintained at 37 °C; gas composition: 95% N_2_, 5% CO_2_) for 40 min. Following hypoxic exposure, the inserts with hippocampal slices or hCMEC/D3 cells were transferred back to starting serum-free plates and grown under standard conditions (37 °C; 5% CO_2_). Nanoparticles, either AOT, AOT-Gd, or PCL and PCL-Gd, as well as carnosic acid (CA) and nanoparticles loaded with CA were administered to the culture medium 30 min before the OGD procedure. All colorimetric or biochemical assays were conducted 24 or 48 h after OGD procedure initiation.

### Propidium Iodide Cell Viability Flow Cytometry

Twenty-four hours after the OGD procedure, the hippocampal organotypic cultures were stained with propidium iodide (PI) as we previously described by Głombik et al. [24] with modifications. Briefly, hippocampal cultures were washed twice with ice-cold HBSS (Gibco, MA, USA) and transferred to 1.5-ml centrifuge tubes containing 500 μl of HBSS. After 5 min of centrifugation (1200 rpm), the cultures were incubated with 500 μl of prewarmed collagenase A solution (2 mg/ml; 30 min at 37 °C; Gibco, NY, USA), centrifuged, and incubated again with 500 μl of prewarmed trypsin (0.05%; 20 min at 37 °C; Sigma-Aldrich, MO, USA). Next, the cultures were stained with a PI solution (100 ng/ml in PBS; Sigma-Aldrich, Germany) for 15 min at room temperature. The hippocampal cells were analyzed using the BD Fluorescence-Activated Cell Sorting (FACS) Canto II System and BD FACS Diva™ v5.0.1 Software (BD Biosciences, USA) in the fluorescence channel for PE (phycoerythrin, red fluorescence). The PI-negative cells were considered to be undamaged and alive, the PI-intermediate cells were considered to be apoptotic, and the PI-positive cells were considered to be dead.

### Lactate Dehydrogenase Assay

A lactate dehydrogenase (LDH) assay was conducted using a Cytotoxicity Detection Kit (Roche, Germany) as previously described [23, 25]. Briefly, 24 or 48 h after OGD, the culture medium was collected, and 50 μL of each sample was placed into a 96-well plate. Then, an equal amount of reagent mixture prepared according to the manufacturer’s instructions was mixed with the samples. After 30 min incubation at 37 °C, the intensity of the red color formed in the colorimetric assay was measured at a wavelength of 490 nm (Infinite® 200 PRO plate reader, Tecan, Zurich, Switzerland) and was proportional to the number of damaged/dead cells.

### Nitric Oxide Release Assay (Nitrite Ion in Solution)

The amount of nitrite ion in solution (NO) was detected as we previously described [26], using a colorimetric Griess reaction under the protocol. An equal volume of the collected culture medium (50 μL), Griess A (0,1% N-1-naphthyl ethylenediamine dihydrochloride) and Griess B (1% sulfanilamide in 5% phosphoric acid; Sigma-Aldrich, St. Louis, MO, USA), was mixed in a 96-well plate. After 5 min incubation at room temperature, the intensity of the formed color was measured at a wavelength of 540 nm (Infinite® 200 PRO plate reader, Tecan, Zurich, Switzerland).

### Enzyme-Linked Immunosorbent Assay (ELISA)

For ELISA assays, the OHC supernatants were collected 24 h or 48 h after the OGD procedure. Moreover, OHCs were lysed using 160 μL of RIPA buffer with Halt™ Protease and Phosphatase Inhibitor Cocktail (Thermo Fisher Scientific, Waltham, MA, USA). Protein isolation was performed, and the total concentration of the protein was assessed using a Pierce™ BCA Protein Assay Kit (Thermo Fisher Scientific, Waltham, MA, USA). Optical density was measured at a wavelength of 562 nm using an Infinite® 200 PRO plate reader (Tecan, Zurich, Switzerland). The levels of interleukin 1β (IL-1β; obtained from Wuhan Fine Biotech Co., Ltd. Wuhan, China), interleukin 6 (IL-6; obtained from Wuhan Fine Biotech Co., Ltd. Wuhan, China), interleukin 4 (IL-4; obtained from Wuhan Fine Biotech Co., Ltd. Wuhan, China), interleukin 10 (IL-10; obtained from Wuhan Fine Biotech Co., Ltd. Wuhan, China), C–C motif chemokine ligand 2 (CCL2; obtained from Wuhan Fine Biotech Co., Ltd. Wuhan, China), C–C motif chemokine ligand 3 (CCL3; obtained from Wuhan Fine Biotech Co., Ltd. Wuhan, China), C–C motif chemokine ligand 5 (CCL5; obtained from Wuhan Fine Biotech Co., Ltd. Wuhan, China), and C-X-C motif chemokine ligand 10 (CXCL10; obtained from Wuhan Fine Biotech Co., Ltd. Wuhan, China) were measured in the collected supernatants. The hypoxia-inducible factor 1α (HIF-1α; obtained from Wuhan Fine Biotech Co., Ltd. Wuhan, China), NFκB p65 Total/Phospho (NFκB p65 Total/Phospho; obtained from Thermo Fisher Scientific, CA, USA), and A20 (Rat Tumor Necrosis Factor Alpha Induced Protein 3(TNFaIP3) or A20 obtained from Wuhan Xinqidi Biological Technology, China) were assessed in the cell lysates isolated from OHCs. All tests were performed using commercially available enzyme-linked immunosorbent assays (ELISA) in accordance with the manufacturer’s protocols. The detection limits were as follows: IL-1β < 18.75 pg/mL, IL-6 < 37.5 pg/mL, IL-4 < 9.375 pg/mL, IL-10 < 18.75 pg/mL, CCL2 < 9.375 pg/mL, CCL3 < 18.75 pg/mL, CCL5 < 9.375 pg/mL, CXCL10 < 18.75 pg/mL, HIF-1α < 4.688 pg/mL, A20 < 10 ng/mL. The inter-assay precision of all ELISA kits was CV% < 12%. The intra-assay precision of all ELISA kits was CV% < 8%. The NFκB p65 Total/Phospho level was measured qualitatively as the relative concentration and normalized to the total protein concentration.

### Materials for Cell Culture

Endothelial cell basal medium (EBM-2) was provided by Lonza Group Ltd. (Basel, Switzerland). Cultrex Rat Collagen I was from R&D Systems (Minneapolis, MN, USA). FluoroBrite DMEM, heat-inactivated fetal bovine serum (FBS), DPBS, penicilin-streptomycin mixture (10,000 U/mL), Trypsin–EDTA solution, HEPES, chemically defined lipid concentrate and human basic fibroblast growth factor (bFGF) were supplied by Gibco (Invitrogen, Paisley, UK). Sulforhodamine B acid chloride and ascorbic acid were purchased from Sigma-Aldrich Chemie GmbH (Germany). Collagen Coating Transwell Inserts (pore size 1 µm) and suitable 24-well plates were obtained from Corning Life Sciences (New York, USA). All materials were used without further purification.

### hCMEC/D3 Cell Culture

Human cerebral microvascular endothelial (hCMEC/D3) cell line (obtained from Sigma-Aldrich Chemie GmbH, Germany), as an in vitro model of BBB was prepared following the procedure described by Łukasiewicz et al. [27]. hCMEC/D3 cells were maintained in 75 cm^2^ flasks pre-coated with 150 µg/ml rat collagen type I (at least 1 h before use and placed in the incubator) in endothelial basal medium-2 (EBM-2) supplemented with 5% (v/v) heat-inactivated FBS, 1% (v/v) penicillin–streptomycin mixture, 1% (v/v) HEPES, 1% (v/v) chemically defined lipid concentrate, 5 µg/ml ascorbic acid, 500 ng/ml hydrocortisone, and 1 ng/ml bFGF (prepared according to manufacturer’s protocol). Cells were cultured at 37 °C under an atmosphere of 5% CO_2_. The culture medium was replaced with fresh medium every 2 days. After reaching 80% confluency, hCMEC/D3 cells were passaged using 0.25% Trypsin/EDTA solution (37 °C, 15–20 min) and centrifugated (900 rpm, 8 min). Next, the pellet was resuspended in fresh EBM-2 medium, counted using a Bürker chamber, and seeded in flasks or plates in suitable density for further experiments. Cells were used for experiments between passages 27 and 35.

### hCMEC/D3 Cell Treatment

hCMEC/D3 cells were seeded at a density of 4.5 × 10^4^ cells/well on Transwell inserts precoated with rat collagen type I (150 µg/ml, 1 h before use in 37 °C) in 24-well plates (Corning, USA) and were cultured for 5 days at 37 °C, 5% CO_2_, and 95% humidity to form confluent monolayer. The culture medium was changed every 2 days and before the treatment. The experiments were performed 5 days after seeding the cells, allowing sufficient time to develop the junctions between cells [27]. After the OGD procedure, the apical chambers (Transwell inserts) were filled with 75 µl or 150 µl of rhodamine-labeled nanoparticles (AOT-ROD/CA and PCL-ROD/CA) and supplemented with EBM-2 medium up to 750 µl. The well under the insert consisted of 750 µl fresh Fluorobrite DMEM, which ensures undisturbed fluorescence measurements. Additionally, rhodamine B alone (diluted in ethanol:water 50% mixture) and 0.015 M NaCl were placed in selected inserts as a control. Plates were incubated at 37 ℃ under standard conditions. At different time points, 50 µl of medium from wells was collected in 96-well black plates and additionally replenished with 50 µl of Fluorobrite medium. Likewise, the content of wells under the inserts was refilled with fresh Fluorobrite medium every time. The penetration ability was determined by measuring fluorescence intensity at the excitation and emission wavelengths of 558 nm and 586 nm, respectively, using an Infinite® 200 PRO plate reader (Tecan, Zurich, Switzerland). All measured fluorescence values were corrected by subtracting the background signal from the pure Fluorobrite medium.

### TEER Measurements

Trans epithelial electrical resistance (TEER, in Ω·cm^2^) was measured every day to confirm the presence of tight monolayer of hCMEC/D3 cells using Millicell ERS-2 Electrical Resistance System (Epithelial Volt–Ohm Meter, Merck Millipore, Burlington, MA, USA). Before the measurements, the electrode was sterilized in 70% ethanol for 15 min. Wells with collagen-coated Transwell inserts without cells were considered a control in TEER measurements. The resistance was calculated by multiplying the Transwell insert’s area with the obtained resistance value. The model of the blood–brain barrier is completely prepared for further studies when the resistance reaches the highest value among all measurements.

### Statistical Analysis

Data were analyzed using the Statistica 14 software (StatSoft Inc., Tulsa, OK, USA). The analysis of variance (one-way ANOVA) and post hoc Duncan’s test for multiple comparisons were used to show statistical significance with assumed *p* < 0.05. The results were obtained from independent experiments carried out under the same conditions and are presented as the mean ± SEM. All graphs were prepared using GraphPad Prism 9.

## Results

### Synthesis and Characterization of Multilayered Nanocarriers of Carnosic Acid

**Fig. 1:**
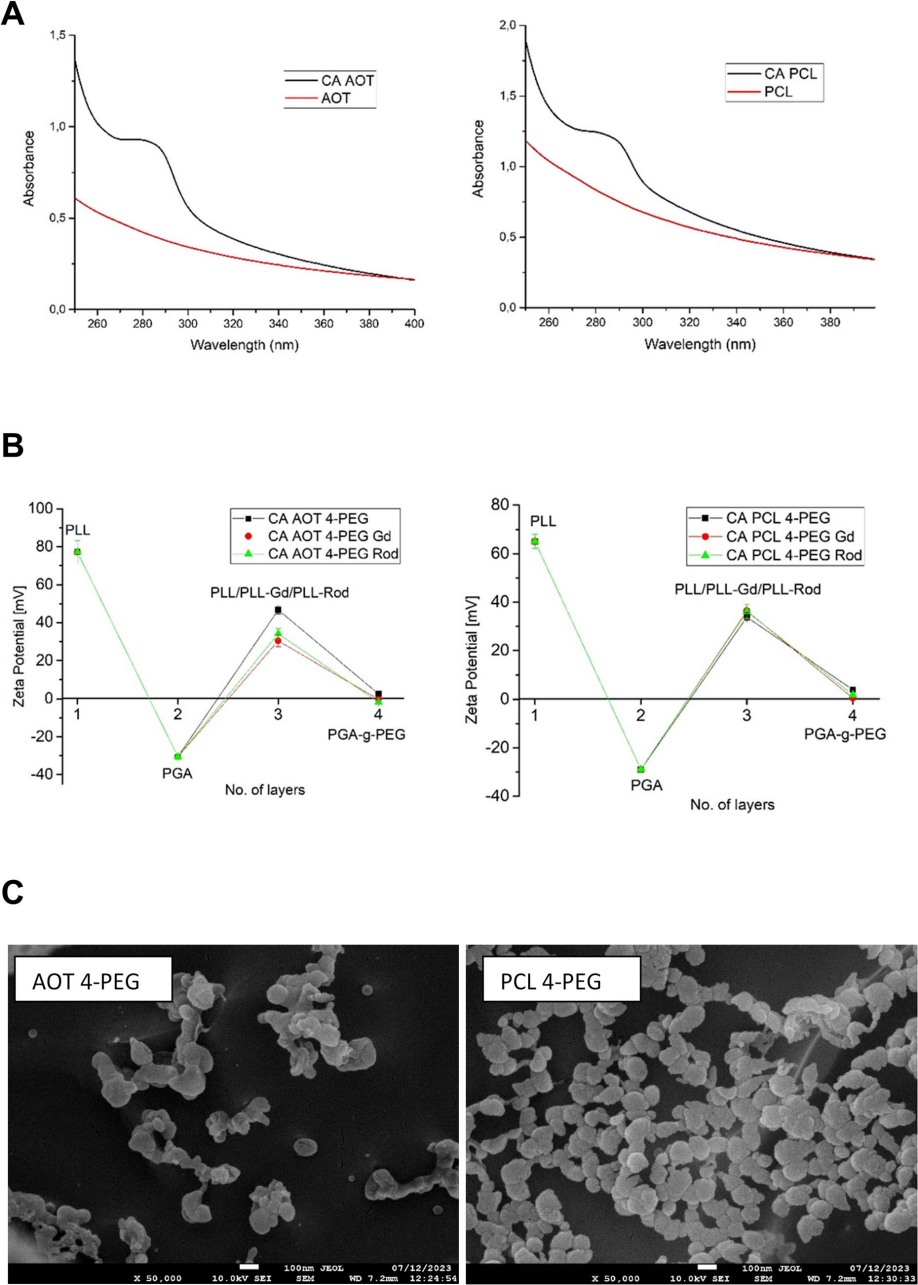
Comparison of UV–vis spectra of empty and carnosic acid-loaded nanocarriers AOT and PCL (**A**). The dependence of nanocarrier zeta potential on the adsorption of consecutive polyelectrolyte layers for AOT and PCL carriers and various shell composition (**B**), example of cryo-SEM images of prepared nanocarriers (**C**)

The multilayered carnosic acid nanocarriers were formulated by encapsulating two types of nanocores (nanoemulsion (AOT) and polymeric (PCL)) containing carnosic acid into multilayer shells using the sequential adsorption of charged nanoobjects method, called layer by layer. The nanocores containing carnosic acid were formed by the spontaneous emulsification solvent evaporation (SESE) method. The average size of formulated nanocores was 80 nm with a polydispersity index (PDI) below 0.2, while the zeta potential value was + 69 ± 12 mV, ensuring electrostatic stabilization of the nanosystem. Comparison of UV–vis spectra of empty and carnosic acid-loaded nanocarriers provided evidence of successful encapsulation of the drug (Fig. 1A). Since carnosic acid is practically not soluble in water, we assumed 100% encapsulation efficiency, and the UV–vis measurements confirmed that assumption. The suspension of nanocores was separated by ultrafiltration (Amicon Ultra MWCO 3000), and drug content in the supernatant was measured, revealing no free drug molecules. The formulated nanocores were encapsulated into a multilayered shell via the layer-by-layer technique, saturation approach, with the following polyelectrolytes: PLL, PGA, PLL-Gd, PLL-Rod, PGA-g-PEG. The procedure was as follows: during continuous stirring, a fixed volume of the nanocores or nanocarriers’ suspension was added to various volumes of oppositely charged polyelectrolyte solution. The zeta potential measurements monitored the layer formation, and the optimal amount of polyelectrolyte was determined based on the saturation technique [15, 20, 28]. The successive deposition of polyelectrolyte layers was repeated until the proper multilayer shell was created. The primary evidence of the formation of the multilayer structure is shown in Fig. 1B, where the typical dependence of nanocarrier zeta potential on the adsorption of consecutive polyelectrolyte layers is presented. Three types of multilayered shells were constructed: simple one based on polyaminoacids PGA/PLL, functionalized with gadolinium complex PGA/PLL-Gd, and functionalized with rhodamine dye PGA/PLL-Rod. All multilayered shells were ended with pegylated-PGA, which allowed the formation of pegylated nanocarriers. The average sizes of formulated multi-layered nanocarriers were 120–200 nm, and the value of zeta potential was around zero, i.e., + 2 ± 2 mV due to the formation of PEG corona. Nanocarriers were stable for up to 2 weeks since we observed no aggregation in the system. An external layer formed with pegylated-PGA provides steric stabilization to the nanosystem. The final carnosic acid concentration in a nanocarrier of both types of cores, including all dilutions during multilayer shell formation, was 18.9 mg/L.

### The Effect of Empty Nanocapsules Without and with Gadolinium on the Cytotoxicity Parameters

To estimate the potential neurotoxicity of empty AOT and PCL nanoparticles without and with gadolinium (AOT, PCL, and AOT-Gd, PCL-Gd, respectively), we assessed their impact on the release of LDH, which is a cell death marker, and NO—a marker of nitroso-oxidative stress in basal condition and after OGD procedure.

**Fig. 2:**
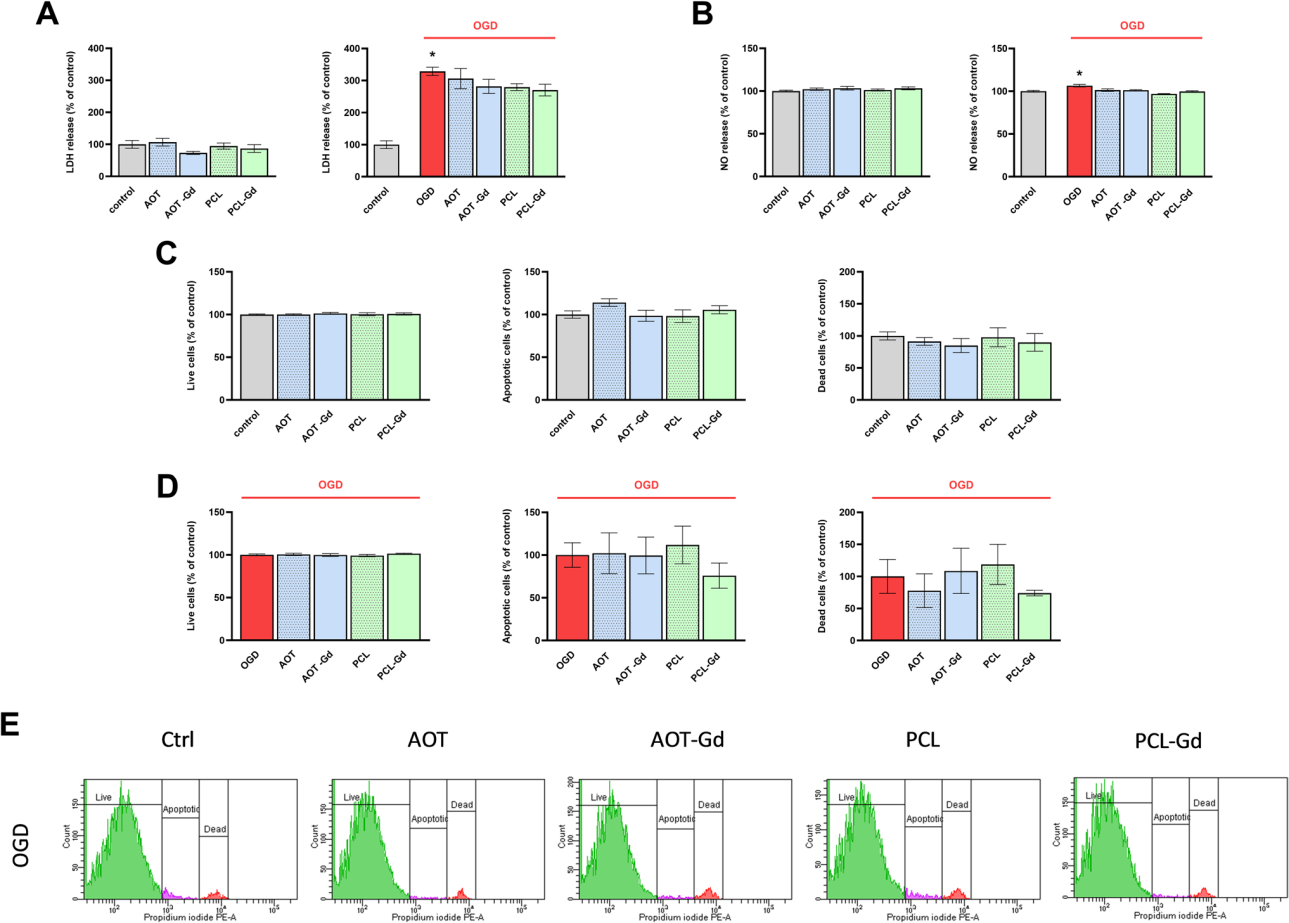
The effect of 100 × diluted nanoparticles on lactate dehydrogenase (LDH) (**A**) and nitric oxide (NO) (**B**) release in control and OGD-treated OHCs. Cultures were pre-treated with nanoparticles: AOT, AOT-Gd, PCL, or PCL-Gd for 30 min. Afterwards, OHCs were subjected to OGD procedure for 40 min and treated again with nanoparticles. The culture supernatants were collected after 24 h and LDH and NO release was measured. The results are expressed as the mean percentages relative to the control ± SEM, *N* = 4–10. The impact of examined nanoparticles on PI uptake in basal (**C**) and OGD-treated (**D–E**) OHCs (assayed by the flow cytometry method). Illustration of the percentage of live cells (PI-negative cells), apoptotic cells (PI-intermediate cells), and dead cells (PI-positive cells) in the examined groups. The results are expressed as the mean percentages relative to the control or OGD ± SEM, *N* = 5–10. Statistical analysis was carried out using one-way analysis of variance (ANOVA) with the Duncan post hoc test to assess the differences between the treatment groups. Significant differences are indicated by *p < 0.05 vs. control

The results 24 h after the OGD procedure showed increased LDH release (*p* = 0.00003) (Fig. 2A). Treatment by NCs did not affect these parameters. Exposure to OGD also elevated the level of NO (*p* = 0.004), while the addition of all types of NCs (AOT, AOT-Gd, PCL, or PCL-Gd) did not modulate this increase in NO release in OHCs (Fig. 2B).

Next, our studies focused on the assessment of NCs on the cell viability in OHCs with the use of propidium iodide staining and flow cytometry analysis. We compared the impact of NCs on the percentage of live, apoptotic, and dead cells (Fig. 2C–E) in normal conditions and after OGD. We did not observe any significant changes in cell viability.

Considering the lack of gadolinium toxicity on the measured parameters and the fact that gadolinium-containing nanoparticles are suitable for MRI detection, for the further set of experiments, we selected nanoparticles with gadolinium.

### The Effect of Empty Nanocapsules with Gadolinium on the Cytotoxicity Parameters and Hypoxia-Inducible Factor 1 Alpha

**Fig. 3:**
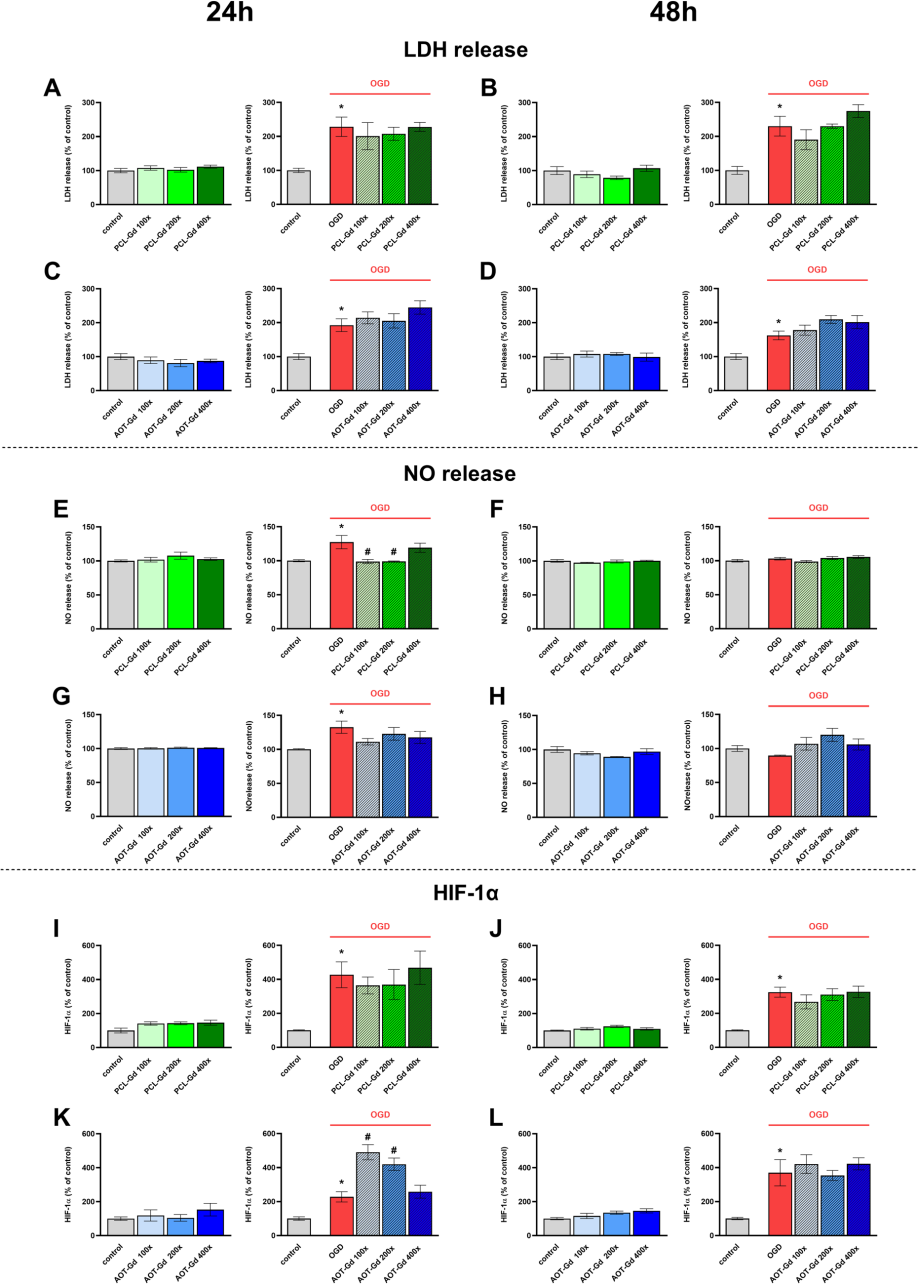
The effect of different concentration (100 × , 200 × , and 300 × diluted) of nanoparticles on lactate dehydrogenase (LDH) (**A–D**) and nitric oxide (NO) (**E–H**) release and on hypoxia-inducible factor 1-alpha (HIF-1α) level (**I–L**) in control and OGD-treated OHCs. Cultures were pre-treated with nanoparticles for 30 min. Afterwards, OHCs were subjected to OGD procedure for 40 min and treated again with nanoparticles. The culture supernatants were collected after 24 or after 48 h and LDH and NO release was measured. The cell lysates were collected after 24 or after 48 h and HIF-1α level was measured and normalized to the total protein concentration. The results are expressed as the mean percentages relative to the control ± SEM, LDH, NO *N* = 6, HIF-1α *N* = 3–6. Statistical analysis was carried out using one-way analysis of variance (ANOVA) with the Duncan post hoc test to assess the differences between the treatment groups. Significant differences are indicated by *p < 0.05 vs. control, ^#^*p* < 0.05 vs. OGD

We studied the effect of AOT-Gd and PCL-Gd in different dilution ranges (100 × , 200 × , and 300 ×) on LDH (Fig. 3A–D) and NO (Fig. 3E–H) release after 24 and 48 h of incubation. In basal condition and after the OGD procedure, none of these tested concentrations of both types of nanoparticles was toxic. However, we reported that after 24 h of incubation, PCL-Gd nanoparticles diluted 100 × and 200 × decreased the OGD-evoked NO production (*p* = 0.010 and *p* = 0.061, respectively).

In the next step, we examined whether Gd-containing nanoparticles affected the level of hypoxia-inducible factor 1-alpha (HIF-1α). OGD procedure elevated the level of HIF-1α as measured after 24 and 48 h following OGD (Fig. 3I–L). The PCL-Gd nanocapsules did not affect the HIF-1α production either after 24 or 48 h of incubation both in basal and after OGD procedure conditions. However, AOT-Gd nanoparticles diluted 100 × and 200 × increased the level of HIF-1α after 24 h of incubation (*p* = 0.0001 and *p* = 0.0018, respectively) in OHCs after OGD (Fig. 3K).

### The Effect of Empty Nanocapsules with Gadolinium on the Pro-inflammatory and Anti-inflammatory Cytokines and Chemokines Release

The inflammatory process and the production of pro- and anti-inflammatory factors play a key role in the time-dependent pattern of ischemia. Moreover, its modulation by additional factors, including the immunogenicity of potential drug carriers, may significantly affect the course and therapeutic success of the ischemia process. Therefore, in the next stage of research, we measured the impact of empty NCs with gadolinium on the pro-inflammatory and anti-inflammatory cytokines and chemokines released in OHC supernatants. Twenty-four hours after the OGD procedure, we noted changes in cytokine balance. A tendency to elevation of IL-1β production, a decrease of IL-4 level (*p* = 0.01), and increased release of IL10 (*p* = 0.004) are demonstrated in Table 1. The OGD procedure did not affect the IL-6 production. Neither the PCL-Gd nor AOT-Gd nanoparticles induced changes in studied cytokines.

**Table 1:** The effect of 100 × diluted nanoparticles on on the pro-inflammatory and anti-inflammatory cytokines: interleukin 1 beta (IL-1β), interleukin 6 (IL-6), interleukin 4 (IL-4), interleukin 10 (IL-10) and chemokines: C–C motif chemokine ligand 2 (CCL2), C–C motif chemokine ligand 3 (CCL3), C–C motif chemokine ligand 5 (CCL5), and C-X-C motif chemokine ligand 10 (CXCL10) release in control and OGD-treated OHCs

	Control			OGD		
	Control	AOT-Gd	PCL-Gd	OGD	AOT-Gd	PCL-Gd
Cytokine						
*IL-1β*	100.0 ± 10.6	76.8 ± 8.6	102.6 ± 7.8	114.5 ± 9.7	88.6 ± 3.8	98.2 ± 13.5
*IL-6*	100.0 ± 5.3	121.2 ± 8.4	78.6 ± 5.4	86.3 ± 3.0	103.6 ± 1.2	77.9 ± 2.5
*IL-4*	100.0 ± 9.3	87.0 ± 7.6	84.2 ± 2.9	70.2 ± 3.3*	79.3 ± 4.5	90.0 ± 0.7
*IL-10*	100.0 ± 4.4	95.9 ± 9.9	117.8 ± 10.0	139.1 ± 7.8*	115.7 ± 3.7	137.6 ± 8.2
Chemokine						
*CCL2*	100.0 ± 7.1	80.4 ± 13.8	129.8 ± 17.3	152.7 ± 12.7*	122.8 ± 7.5	154.7 ± 16.0
*CCL3*	100.0 ± 12.9	69.3 ± 13.3	110.0 ± 6.9	183.5 ± 13.2*	154.4 ± 6.4	196.9 ± 7.2
*CCL5*	100.0 ± 11.7	76.0 ± 5.4	95.0 ± 6.7	98.0 ± 4.3	99.2 ± 3.7	112.6 ± 1.3
*CCL10*	100.0 ± 13.7	92.3 ± 9.3	133.8 ± 6.6	212.0 ± 27.4*	141.1 ± 3.5	233.6 ± 41.1

Cultures were pre-treated with nanoparticles for 30 min. Afterward, OHCs were subjected to the OGD procedure for 40 min and treated again with nanoparticles. The supernatants were collected after 24 h and cytokine and chemokine levels were measured. The results are expressed as the mean percentages relative to the control ± SEM, *N* = 3–6. Statistical analysis was carried out using one-way analysis of variance (ANOVA) with the Duncan post hoc test to assess the differences between the treatment groups. Significant differences are indicated by *p < 0.05 vs. control

Moreover, in the case of chemokines, we observed an elevated level of CCL-2, CCL3, and CXCL10 in OGD-treated cultures (*p* = 0.016, *p* = 0.0009, *p* = 0.017, respectively) (Table 1). On the other hand, the production of CCL5 was not changed. No effect of incubation with examined nanoparticles was revealed in any of the investigated chemokine levels.

Thus, these observations suggest that empty nanocapsules do not modulate the levels of cytokines and chemokines, both under basal conditions and after OGD induction in OHCs.

### The Effect of Nanoparticles on the Permeability in the Experimental Blood–Brain Barrier Model

In the next part of the research, the previously tested NCs were used as a system for delivering compounds with neuroprotective potential under conditions of experimental ischemia. According to our recently published data [13], we selected carnosic acid (CA). First, we examined the effect of CA-loaded PCL-ROD (PCL-ROD/CA) and AOT-ROD (AOT-ROD/CA) nanoparticles on the permeability of the cell layer of hCMEC/D3 cells—a model of BBB.

**Fig. 4:**
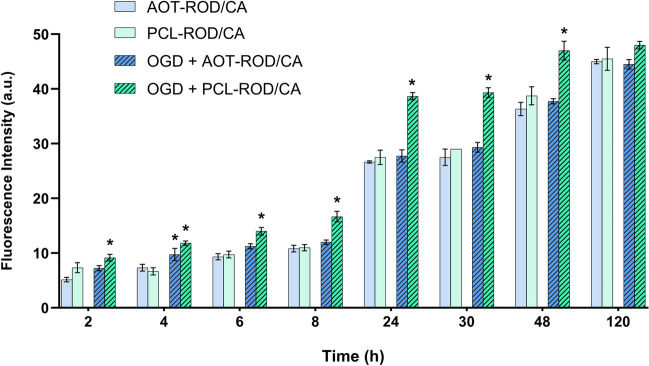
Penetration of various types of rhodamine-labeled (100 × diluted CA-loaded AOT-ROD or PCL-ROD) nanocapsules through the blood–brain barrier under basal conditions and after the OGD procedure expressed by fluorescence intensity in hCMEC/D3 cells (transwell pore—1 mm). The results are expressed as the mean ± SEM, *N* = 2–6. Statistical analysis was carried out using one-way analysis of variance (ANOVA) with the Duncan post hoc test to assess the differences between the treatment groups. Significant differences are indicated by * *p* < 0.05 vs. control at the same time point

We observed the time-dependent penetration ability of all types of examined nanoparticles (AOT-ROD, PCL-ROD) loaded with CA measured as fluorescence intensity in basal and after OGD procedure conditions. However, the penetration ability was higher after the OGD procedure in particular for PCL-ROD/CA (Fig. 4).

Since PCL-ROD/CA-loaded nanoparticles showed greater effectiveness in penetrating the BBB under OGD conditions, we introduced this type of NCs as theranostic drug carriers in our further research.

### The Impact of Free Carnosic Acid and Carnosic Acid Loaded in Nanoparticles on the Cytotoxicity Parameters

**Fig. 5:**
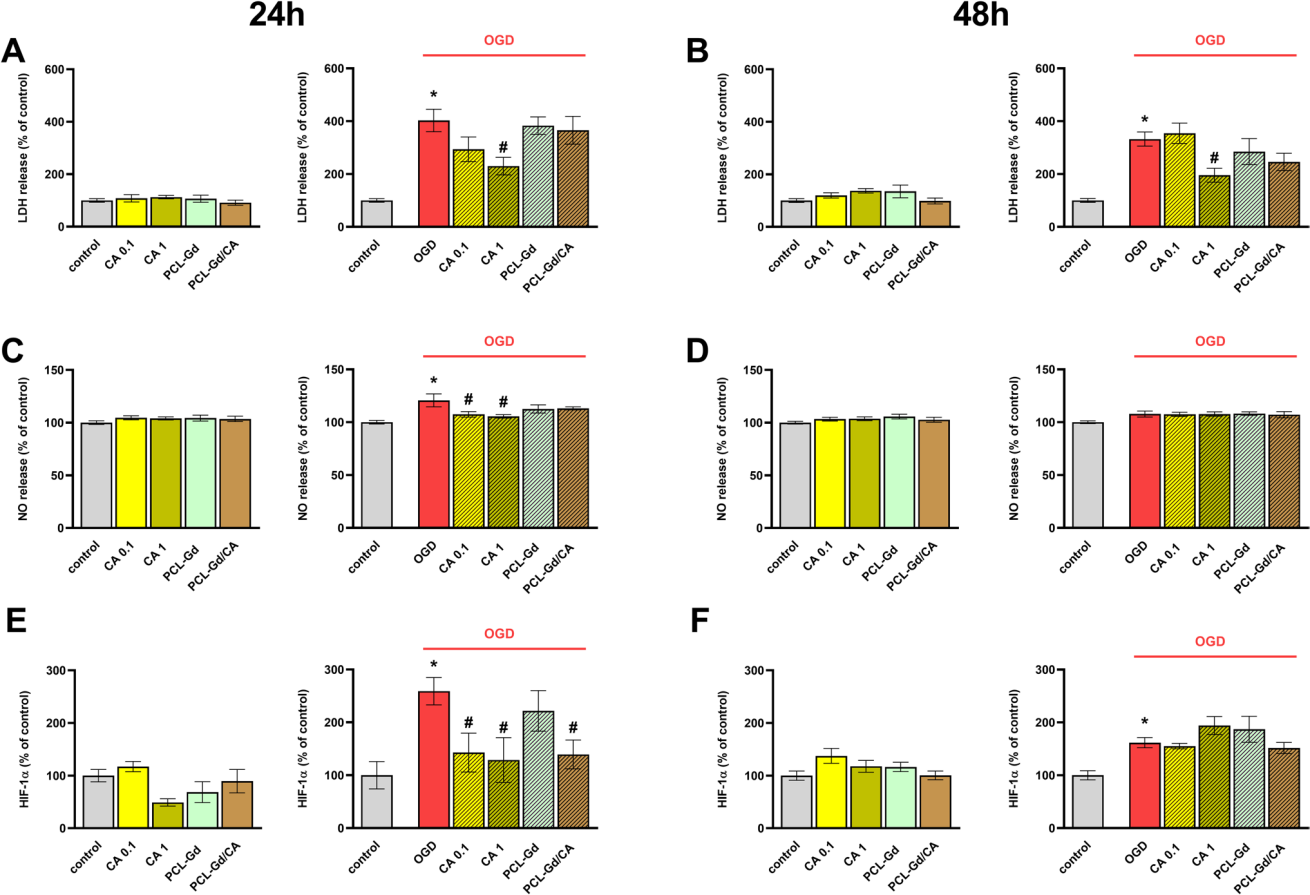
The effect of 0.1 and 1 μM carnosic acid (CA), empty 100 × diluted PCL-Gd nanoparticles, and 100 × diluted CA-loaded PCL-Gd nanoparticles (PCL-Gd/CA) on lactate dehydrogenase (LDH) (**A–B**), nitric oxide (NO) release (**C–D**), and hypoxia-inducible factor 1-alpha (HIF-1α) level (**E–F**) in control and OGD-treated OHCs. Cultures were pre-treated with CA or nanoparticles for 30 min. Afterwards, OHCs were subjected to OGD procedure for 40 min and treated again with CA or nanoparticles. The culture supernatants were collected after 24 (**A**, **C**) or 48 (**B**, **D**) hours and LDH and NO release was measured. The cell lysates were collected after 24 (**E**) or 48 (**F**) hours and HIF-1α level was measured and normalized to the total protein concentration The results are expressed as the mean percentages relative to the control ± SEM, *N* = 3–6. Statistical analysis was carried out using one-way analysis of variance (ANOVA) with the Duncan post hoc test to assess the differences between the treatment groups. Significant differences are indicated by *p < 0.05 vs. control, ^#^*p* < 0.05 vs. OGD

The next part of the experiments involved free CA (at the concentration of 0.1 and 1 µM) and CA-loaded PCL-Gd nanoparticles (PCL-Gd/CA). In time-dependent studies, we did not observe any cytotoxic effect of either free CA or PCL-Gd/CA in basal conditions. Importantly, we reported that free CA in the concentration of 1 µM decreased the OGD-induced elevated amount of LDH released into the medium after 24 and 48 h of incubation (*p* = 0.015, *p* = 0.043, respectively) (Fig. 5A–B).

Moreover, 0.1 and 1 µM free CA after 24 h of incubation diminished the release of NO after the OGD procedure (*p* = 0.036, *p* = 0.020, respectively) (Fig. 5C).

Next using ELISA assays, we examined the effect of free CA and PCL-Gd/CA on the level of HIF-1α in OHC’s lysates. The concentration of 0.1 and 1 µM free CA and PCL-Gd/CA reduced the level of HIF-1α (*p* = 0.030, *p* = 0.021, *p* = 0.030, respectively) after 24 h of incubation (Fig. 5E).

### The Effect of Free Carnosic Acid and Carnosic Acid Loaded in Nanoparticles on the Release of Pro- and Anti-inflammatory Cytokines

Next, we study the impact of free CA and PCL-Gd/CA on the release of pro- (IL-1β, IL-6) and anti- (IL-4, IL-10) cytokine release in basal and OGD-affected OHCs. After 48 h of incubation, we observed suppression of OGD-evoked IL-1β release after 0.1 and 1 µM CA as well as PCL-Gd/CA treatment (*p* = 0.0060, *p* = 0.0065, *p* = 0.0031, respectively) (Table 2). On the other hand, the level of IL-1β after 24 h of incubation was not altered.

**Table 2:** The effect of 0.1 and 1 μM carnosic acid (CA), empty 100 × diluted PCL-Gd nanoparticles, and 100 × diluted CA-loaded PCL-Gd nanoparticles (PCL-Gd/CA) on interleukin 1 beta (IL-1β), interleukin 6 (IL-6), interleukin 4 (IL-4) in control (A) and OGD-treated (B) OHCs

A	Control				
	Control	CA 0.1	CA 1	PCL-Gd	PCL-Gd/CA
24 h					
*IL-1β*	100.0 ± 3.5	111.1 ± 8.8	103.2 ± 7.0	94.8 ± 3.9	119.1 ± 8.8
*IL-6*	100.0 ± 7.9	96.1 ± 4.9	90.7 ± 5.4	79.7 ± 1.3	81.3 ± 3.6
*IL-4*	100.0 ± 8.3	98.4 ± 8.7	79.3 ± 2.4	80.0 ± 4.1	92.4 ± 10.6
48 h					
*IL-1β*	100.0 ± 0.6	113.1 ± 7.4	120.2 ± 14.0	98.3 ± 5.2	93.2 ± 5.1
*IL-6*	100.0 ± 7.7	121.0 ± 8.7	109.6 ± 11.0	86.6 ± 2.5	90.7 ± 7.4
*IL-4*	100.0 ± 3.2	104.9 ± 6.6	107.7 ± 8.9	93.5 ± 4.7	104.7 ± 8.0
B	OGD				
	OGD	CA 0.1	CA 1	PCL-Gd	PCL-Gd/CA
24 h					
*IL-1β*	109.3 ± 10.1	118.0 ± 14.8	107.5 ± 6.1	115.2 ± 9.5	101.6 ± 2.6
*IL-6*	96.2 ± 8.8	105.5 ± 9.3	107.1 ± 8.4	87.0 ± 9.2	96.4 ± 11.0
*IL-4*	86.1 ± 3.9	112.2 ± 9.2	154.1 ± 21.2^#^	112.8 ± 13.0	109.7 ± 9.5
48 h					
*IL-1β*	148.2 ± 15.9*	90.4 ± 4.4^#^	92.2 ± 5.7^#^	117.8 ± 21.4	84.5 ± 1.9^#^
*IL-6*	114.0 ± 11.3	92.2 ± 6.5	120.9 ± 9.9	86.4 ± 9.0	105.5 ± 4.0
*IL-4*	101.8 ± 3.9	139.1 ± 6.0^#^	129.3 ± 16.0^#^	108.7 ± 3.5	97.7 ± 4.0

Cultures were pre-treated with CA or nanoparticles for 30 min. Afterwards, OHCs were subjected to OGD procedure for 40 min and treated again with CA or nanoparticles. The supernatants were collected after 24 or 48 h and cytokine levels were measured. The results are expressed as the mean percentages relative to the control ± SEM, *N* = 4–6. Statistical analysis was carried out using one-way analysis of variance (ANOVA) with the Duncan post hoc test to assess the differences between the treatment groups. Significant differences are indicated by *p < 0.05 vs. control, ^#^*p* < 0.05 vs. OGD

We noticed also an increase in the release of IL-4 caused by 1 µM free CA after 24 h of incubation (*p* = 0.0043) and by 0.1 and 1 µM doses of CA after 48 h of incubation (*p* = 0.0013, *p* = 0.011, respectively) (Table 2). The level of IL-6 was not changed in any of the time points in basal and after OGD procedure conditions in OHCs.

### The Effect of Free Carnosic Acid and Carnosic Acid Loaded in Nanoparticles on the Selected Intracellular Pathways

To shed more light on the intracellular mechanisms of protective and anti-inflammatory potential of CA-free and PCL-Gd/CA, we investigated some signaling pathways in OHCs after experimental OGD.

**Fig. 6:**
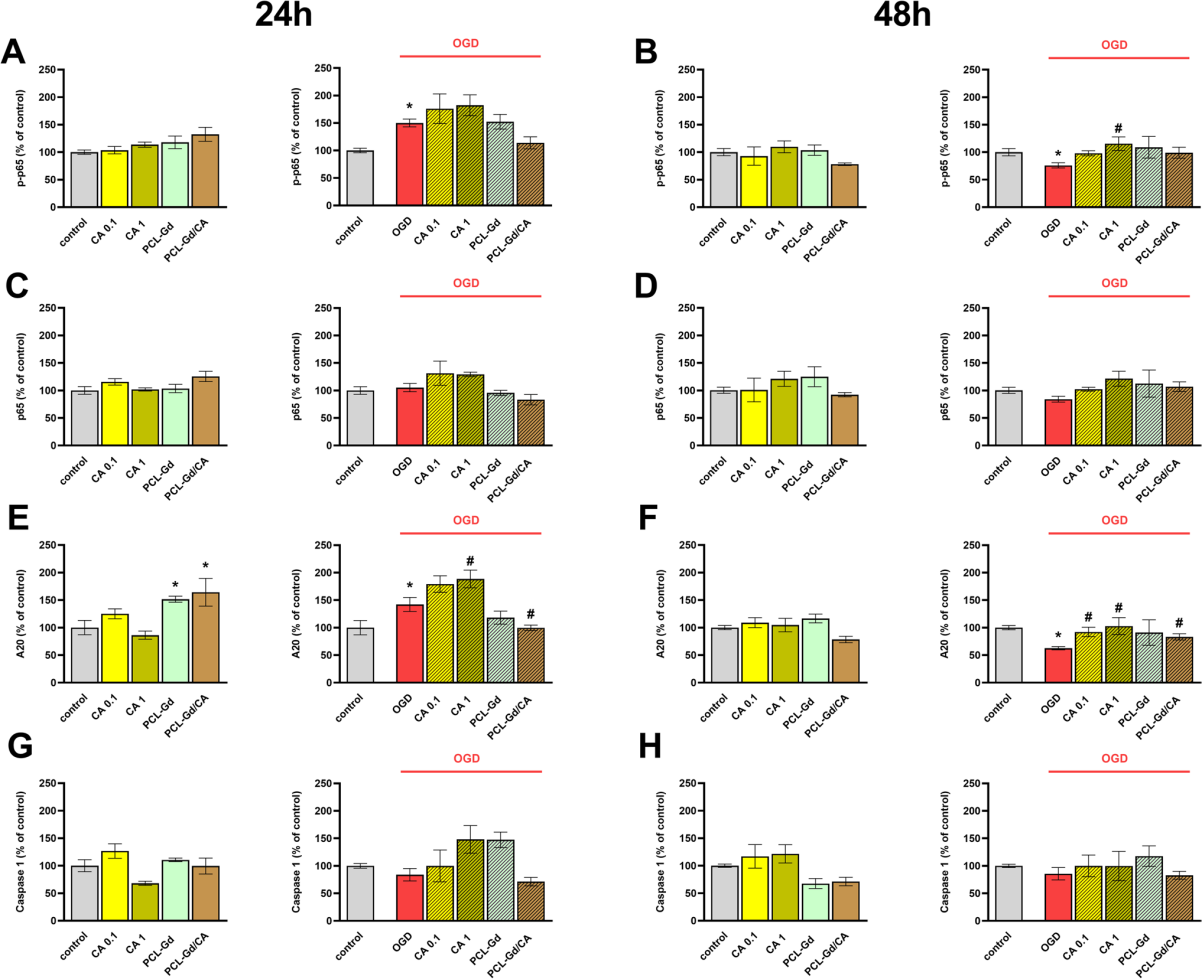
The effect of 0.1 and 1 μM carnosic acid (CA), empty 100 × diluted PCL-Gd nanoparticles and 100 × diluted CA-loaded PCL-Gd nanoparticles (PCL-Gd/CA) on p-p65 (**A–B**), p65 (**C–D**), A20 (**E–F**), and caspase 1 (**G–H**) level in control and OGD-treated OHCs. Cultures were pre-treated with CA or nanoparticles for 30 min. Afterwards, OHCs were subjected to OGD procedure for 40 min and treated again with CA or nanoparticles. Cell lysates were collected after 24 or 48 h and apropiate protein levels were measured. The results are expressed as the mean percentages relative to the control ± SEM, *N* = 3–6. Statistical analysis was carried out using one-way analysis of variance (ANOVA) with the Duncan post hoc test to assess the differences between the treatment groups. Significant differences are indicated by *p < 0.05 vs. control, ^#^*p* < 0.05 vs. OGD

We examined the effect of free CA and PCL-Gd/CA on the level of nuclear factor kappa-light-chain-enhancer of activated B cells (NF*κ*B) signaling. We did not find the impact of CA and PCL-Gd/CA on the NFkB signaling after 24 and 48 h of incubation in basal conditions in OHCs. However, the evoked by OGD increase of p-p65 phosphorylation level after 24 h was tender to diminish after PCL-Gd/CA treatment (Fig. 6A). The concentration of 1 µM CA increased the level of p-p65 (*p* = 0.006) after 48 h of incubation (Fig. 6B). Total level of p65 was not changed in any of the time points (Fig. 6C–D).

Further, we examined the effect of free CA or PCL-Gd/CA on the level of A20. We noticed an increased level of A20 caused by 1 µM CA and decreased level of A20 caused by PCL-Gd/CA after 24 h of incubation (*p* = 0.026, *p* = 0.046, respectively) (Fig. 6E). The concentration of 0.1 and 1 µM CA and PCL-Gd/CA increased the level of A20 (*p* = 0.017, *p* = 0.027,* p* = 0.019, respectively) after 48 h of incubation (Fig. 6F).

NF-κB promotes the transcription of inflammasome 3 (NLRP3). Thus, next, we measured the level of Casp1, which contributes as the main component of the NLRP3 platform, to the production and secretion of pro-inflammatory cytokines mainly IL-1β and IL-18. However, the level of Casp1 was not changed at any of the time points after the OGD procedure. Moreover, free CA or PCL-Gd/CA did not affect the measured level of Casp1 (Fig. 6G–H).

## Discussion

This study demonstrated that the newly designed gadolinium-containing nanoparticles are not toxic to organotypic hippocampal cultures (OHCs) and do not affect detrimental effects of experimental ischemia carried out by oxygen–glucose deprivation (OGD) on the viability of the hippocampal cells. The nanoparticles are also neutral to the course of the inflammatory process accompanying the phenomenon of ischemia. Importantly, they possess the ability to cross artificial BBB. Next, we showed the anti-inflammatory potential of PCL-Gd carnosic acid-loaded nanoparticles, expressed as decreased OGD-induced HIF-1α and IL-1β levels. Our molecular studies suggest a complex mechanism of this nanoformulation on ischemia-related neuroinflammation in OHCs, including anti-inflammatory protein A20 stimulation and moderate attenuation of the NFκB signaling pathway. It should be emphasized here that although the biocompatibility of polymeric nanoparticles as potential drug nanocarriers is routinely examined, their interaction with inflammatory processes remains poorly recognized. This problem merits attention since neuroinflammation plays a crucial role in pathomechanisms of the late phase of ischemic stroke.

Polymeric nanoparticles based on polyethylene glycol (PEG), polylactic-co-glycolic acid (PLGA), and polylactic acid (PLA), among others, are regarded as promising drug delivery systems due to their ability to cross biological barriers, capacity to convey a wide array of therapeutic compounds, low toxicity, and fast elimination [29]. Moreover, they are suitable nanotheranostics components for diagnosing and treating brain disorders, including ischemic stroke. Recently, we demonstrated that the layer-by-layer approach allows the formulation of nanoemulsion core nanocapsules with the size c.a. 100 nm containing SPIONs in the capsule shell [27]. The same methodology was used to synthesize nanocarriers (nanoemulsion or polycaprolactam core-based) with poly-l-lysine/Gd complex as a component of the polyelectrolyte shell [19]. These capsules exhibited no toxic effect on human peripheral blood mononuclear cells. On the other hand, gadolinium ions were used to crosslink alginate nanogels to achieve MRI-traceable nanocarriers [30]. The successful approach of designing nanocarriers with incorporated MRI-sensitive, Gd-containing probes able to cross BBB, based on poly (n-butyl cyanoacrylate) nanoparticles, was presented [31]. Recently, we found that the AOT and PCL polymeric nanocarriers affected the viability of human neuroblastoma SH-SY5Y cells only when used in the highest concentrations, and the presence of two layers labeled with gadolinium does not increase their toxicity (unpublished data). However, when evaluating the biocompatibility of innovative nanoformulations for the diagnosis and treatment of ischemic stroke and other neurodegenerative diseases, it is necessary to consider not only their potential and direct toxicity but also their influence on neuroinflammatory processes, in the induction and course of which both glial and neuronal cells participate.

In numerous ischemia-related studies, OGD has been widely used as an in vitro stroke model with various cellular types from different species. As comprehensively reviewed by [32], different cellular models from rat, human, mouse, bovine, and porcine brain endothelial cells have been used in reports of OGD experiments to mimic as closely as possible the pathological conditions of the vascular system in the ischemic brain. Most of the studies in which experiments performed with human brain endothelial cells have used the primary culture of human cerebromicrovascular endothelial cells (HCECs) or immortalized human brain capillary endothelial cells (hCMEC/D3) as used in our study. The primary brain capillary endothelial cells have the closest similarity to the BBB in vivo phenotype and exhibit excellent characteristics of the BBB at early passages. However, they are time-consuming and costly to generate, are easily contaminated by other neurovascular unit cells (losing their BBB characteristics over passages), rapidly undergo senescence after a limited number of divisions, and require a high level of technical skill to perform their extraction from brain tissue. On the other hand, immortalized lines allow the BBB characteristics to be maintained over many passages, permit the formation of functional barriers, and are amenable to numerous molecular interventions.

In the present study, we employed the OGD model in OHCs, where the integrity of local neuronal circuits and the mutual connection of neurons, glial cells, and extracellular matrix is well preserved [33]. Despite the absence of blood flow in OHCs, microvessels were present and able to respond to angiogenic stimuli like acidosis or hyperthermia [34]. Moreover, the presence of all cell types and their interactions, TJ preservation between endothelial cells and BBB carriers and transporters, and the presence of all cell types and their interactions provide OHCs a complete ex vivo model of the neurovascular unit, although in the absence of blood flow [35]. In agreement with a previous study, the OGD procedure evoked cell damage as revealed by lactate dehydrogenase (LDH) release, decreased cell viability, and enhanced levels of nitric oxide (NO)—a marker of nitroso-oxidative stress [22]. On the other hand, we found that empty AOT and PCL nanocapsules showed no toxic effects on OHCs in basal and OGD conditions, verified also by using propidium iodide and fluorescence-activated cell sorting. The lack of effect of empty AOT and PCL nanocapsules with and without gadolinium on the viability of OHC confirms their safety and usefulness for serving as drug nanocarriers. Furthermore, PCL-Gd nanoparticles attenuated OGD-induced nitric oxide production, 24 h after OGD, but this effect did not translate into their neuroprotective or neurotoxic activity. Notably, NO is regarded as a Janus molecule in the neuronal death/survival mechanisms since, in physiological concentrations, it is neuroprotective, while at higher concentrations, it shows neurotoxicity [36].

All in all, the results of our study confirm the view that polycaprolactam-based nanocarriers show excellent biocompatibility [37]. More importantly, also Gd-containing AOT and PCL nanocapsules were devoid of toxicity, suggesting that both nanoparticle types may be suitable for designing Gd-containing theranostics. However, this suggestion should be treated with caution because the concentration of Gd in our nanoformulation was lower than that in marketed MRI contrast agents, and we did not evaluate the neurotoxicity of Gd alone in our experimental settings. Gd3 + was reported to be highly neurotoxic even at low doses (10^−7^ M), and due to cumulation in brain tissue, it is likely to achieve locally toxic concentrations [38–41]. Other authors reported that gadolinium oxide nanoparticles had toxic effects on human endothelial HUVECs inducing lipid peroxidation, mitochondrial dysfunction, and autophagy modulation [42].

Although both AOT and PCL nanoparticles display comparable biocompatibility, we noticed that PCL nanocapsules penetrated artificial BBB faster than AOT nanocapsules. Therefore, PCL as an essential component of nanotheranostic seems to be more suitable and can represent a novel approach for enabling targeted therapeutic delivery to the brain.

Differences between AOT and PCL nanoparticles were also revealed in terms of their effects on OGD-induced neuroinflammation. Notably, it was found that AOT but not PCL nanoparticles increased hypoxia-inducible factor 1 (HIF-1α). This is a significant finding as it has been firmly established that hypoxia/ischemia rapidly induces the expression of HIF-1α, a subunit of the heterodimeric transcription factor HIF-1. This factor is highly sensitive to changes in oxygen levels through degradation and/or heterodimerization of its subunits, consequently modulating the expression of various genes. Generally, HIF-1 is crucially involved in neuronal death/survival, neuroinflammation, blood–brain barrier modulation, angiogenesis, and glucose metabolism [43]. Therefore, the observed alteration in HIF-1α level after the OGD procedure may have functional significance since activation of this transcription factor during preconditioning promotes cell survival in OGD hippocampal injury [44]. It was also reported that upregulation of the HIF-1α protein triggers mitochondrial autophagy in primary cortical cell cultures exposed to OGD, thereby increasing neuronal survival [45]. On the contrary, other findings showed in an in vitro model of ischemia that the deletion of the HIF-1 gene in neurons increased their mortality, and the use of a stabilizer of HIF-1 heterodimers—compound DMOG, reduced cell death [46]. Equally important are the results from in vivo studies in a mouse model of neonatal ischemia, where 2ME2 (HIF-1 inhibitor) exhibited a neuroprotective effect, diminishing brain edema, and BBB permeability [47]. These contrasting observations underscore the need for further research on this issue.

Of particular interest, HIF-1α has been shown to significantly influence ischemic brain damage by modulating the time-dependent inflammatory response [48]. The involvement of non-neuronal cell populations, including microglia, astrocytes, and leukocytes, in neuroinflammation induced by OGD is well-documented [49]. In acute cerebral ischemia, neuroinflammation mediated by glia may promote ischemic injury, resulting in neuronal death, but on the contrary, neuroinflammation may also play a beneficial role in long-term tissue repair [50]. The role of microglia or astrocytes in this process, whether protective or damaging, determines the panel of mediators, cytokines, and chemokines released depending on the stage of ischemia/reperfusion [51]. The increased proinflammatory cytokine and chemokine levels in OHCs under the influence of OGD align with the results of our previous study, where we determined the impact of OGD on the levels of various pro- and anti-inflammatory cytokines (IL-1β, IL-6, IL-4, IL-10) and chemokines (CCL2, CCL3, CXCL10) in OHCs [22]. Changes in cytokine levels indicate a complex network of events that occur under the influence of OGD procedure in OHCs, leading to homeostasis disturbances in which microglial activation is crucial [22].

Of note, in the present research, the empty nanocapsules did not affect the mediators induced by OGD, which may suggest their usefulness as theranostic carriers in neuroinflammatory-based diseases. So far, biosafety of some polymeric nanoformulations regarding neuroinflammatory processes has been postulated. Thus, poly(lactic-co-glycolic) acid (PLGA)- and l-tyrosine polyphosphate-based nanoparticles did not induce the release of the proinflammatory cytokines like TNF-α, IL-1β, or IL-6 from microglial cells, suggesting that they might be helpful for the delivery of imaging agents and drugs to the sites of neuroinflammation [52]. Indeed, some nanoparticles have been reported to be useful carriers for anti-inflammatory drugs to inhibit the overactivation of microglia and inflammation [53]. On the other hand, regarding gadolinium-containing MRI contrast agents, it has been reported that gadopentetate but not gadobutrol induced cell death in mouse OHCs, and that process was aggravated in an inflammatory milieu [54].

As we mentioned, the designed theranostic nanocarriers showed different experimental BBB penetration abilities in the experimental ischemia in the OHCs model. At the same time, we observed different nanoparticle effects on HIF-1α induction, which, in the light of ambiguous literature data regarding the interpretation of this phenomenon, prompted us to choose PCL as an essential component of nanotheranostic. They appeared also more suitable than AOT, for encapsulation of carnosic acid. Hence, we demonstrated that CA alone diminished LDH release evoked by the OGD procedure and, in a time-dependent manner, alleviated NO production in OHCs exposed to ischemia/reperfusion. Moreover, free CA decreased HIF-1α level 24 h after OGD. Simultaneously, the OGD-induced IL-1β level elevation was diminished by CA, while the release of IL-4 was enhanced 48 h after this procedure. Importantly, PCL-Gd/CA nanoparticles also decreased HIF-1α and IL-1β levels in OHCs exposed to experimental ischemia/reperfusion. These results suggest that CA both in free form and encapsulated in PCL-Gd nanoparticles displays anti-inflammatory activity.

Consequently, to shed more light on the potential intracellular mechanism of anti-inflammatory action of free and CA-loaded nanoparticles in OHCs, we explored their time-dependent impact on some intracellular pathways. In this context, the nuclear transcription factor kappa B (NF-κB) deserves special attention because it is the primary regulator of ischemic injury severity. Reducing NF-κB activation can decrease inflammation after cerebral ischemia and alleviate brain injury [55]. NF-κB promotes inflammation by regulating the expression of a series of inflammation-related genes, which can inhibit NF-κB directly and inhibit NF-κB-mediated signals and inflammatory cytokines [56], preventing neuroinflammation [57]. We found enhanced p-p65 subunit phosphorylation 24 h after OGD and a tendency to attenuate the phosphorylation after PCL-Gd/CA treatment. Although the NF-κB can increase the transcription of inflammasome NLRP3 and inflammatory cytokines, such as IL-1β, we did not find the impact of free CA and PCL-Gd/CA nanoparticles on the caspase-1 level, the crucial subunit of NLRP3 platform. However, the most spectacular observation was that nanoformulations affected the level of A20—an inhibitor of p-p65 phosphorylation in a time-dependent manner. Specifically, CA and PCL-Gd/CA had opposite effects on the A20 level at 24 h, but both of them increased the level of A20 at 48 h after OGD. This is an intriguing finding because neuroinflammation is linked with the late phase of ischemic stroke, and the A20 protein has a potent anti-inflammatory effect, while the inhibition of A20 leads to a more robust inflammatory response after stroke [58]. Moreover, the A20 protein may inhibit the activation of the NLRP3 pathway by inhibiting NF-κB and then inhibit the expression of IL-1β, IL-18, and other inflammatory cytokines in the ischemic cortex [59]. Nevertheless, it should be mentioned that in addition to its role in regulating NF-κB signaling, A20 also acts as a potent inhibitor of cell death in many cell types. In general, A20 is a multifunctional ubiquitin-editing enzyme that could—depending on context and target protein—act as an E3 ligase, participate in ubiquitin binding, deubiquitylate target proteins, or concurrently perform multiple ubiquitin-editing functions on target proteins.

However, in the context of our study, the most important is the observation that A20 is a critical player in the immune system, limiting inflammatory reactions also by impact on the JNK and JAK2-STAT3/A20 signalling [60–62]. By regulating these pathways, A20 influences various cellular processes, enlightening us about its role in cell survival, proliferation, and autophagy. More recently, the pathogenic role of A20 in human inflammation was definitively established by discovering an immunologic disease caused by dominantly inherited hypomorphic A20 mutations (HA20) [63]. Thus, based on available data and our results, it may be suggested a beneficial modulatory effect of CA, both in free form and in nanocapsules, on the activation of the NF-κB inhibitor A20, and in this way, its anti-inflammatory effect.

A few limitations of this study should be considered. First, although the concentrations of Gd used in the nanoformulation did not induce side effects, it cannot be ruled out that higher concentrations are needed to use them as MRI contrast agents. Therefore, the obtained results require confirmation in in vivo preclinical models of ischemia. Moreover, our observations regarding the beneficial anti-inflammatory effects are a correlative, not mechanistic, study.

Finally, our nanoparticles must be specifically and efficiently delivered to the brain. Therefore, the identification of brain-specific ligands that could be used in the development of brain-targeted nanoparticles is also a key aspect. Such ligands can specifically target nanoparticles to brain tissue, avoiding non-specific interactions in other compartments, reducing off-target effects and consequently increasing the bioavailability of dispersed agents. Therefore, to improve the targeted delivery, after crossing the blood–brain barrier, it is important to target CA-loaded nanoparticles to specific cells in the brain parenchyma. In this regard, various strategies have been developed to investigate specific recognition by targeting ligands of different brain-resident cells, such as neurons (aptamer C4-3, neurotensin, Tet-1, RVG, and IKRG peptides), astrocytes (aquaporin-4, D4, and bradykinin B2 antibodies), oligodendrocytes (NG-2 antibody and biotinylated DNA aptamer coupled to streptavidin core Myaptavin-3064), microglia (CD11b antibody), neural stem cells (peptides QTRFLLH, VPTQSSG, and NFL-TBS.40–63) and to BBB endothelial cells (transferrin and insulin proteins and choline) [64]. Based on the abovementioned data, in the next stage of our research, we will functionalize nanoparticles in order to better deliver them to selected brain cells.

In conclusion, this study provided ex vivo observations that the newly designed AOT and PCL nanoparticles, even with gadolinium, were devoid of toxicity in organotypic hippocampal cultures in basal and experimental ischemia conditions. Moreover, they penetrated the experimental blood–brain barrier and did not induce changes in the immune response, assessed by synthesizing pro- and anti-inflammatory markers during ischemia/reperfusion. As a follow-up, we observed that carnosic acid in free and theranostic nanoformulations enhanced the level of A20—an inhibitor of p65 phosphorylation in OHC exposed to OGD, which points to its potential anti-inflammatory properties. Based on the current research results, PCL-Gd/CA nanoformulation seems a promising candidate for designing nanotheranostics for treating ischemic stroke, but this assumption needs to be verified in further models of this brain disorder.

## Data Availability

Data will be made available on request.
